# Inter-Individual Variation and Cardioprotection in Anthracycline-Induced Heart Failure

**DOI:** 10.3390/jcm10184079

**Published:** 2021-09-09

**Authors:** Nadine Norton, Raegan M. Weil, Pooja P. Advani

**Affiliations:** 1 Department of Cancer Biology, Mayo Clinic, Jacksonville, FL 32224, USA; Weil.Raegan@mayo.edu; 2 Department of Hematology and Oncology, Mayo Clinic, Jacksonville, FL 32224, USA; Advani.Pooja@mayo.edu

**Keywords:** cardiomyopathy, chemotherapy, pharmacogenetics, cardiotoxicity, risk variants, doxorubicin, Adriamycin, TRPC6, CBR3

## Abstract

Anthracyclines are one of the most widely used and effective chemotherapies in oncology, but their most important side effect is the cumulative, dose-related cardiotoxicity leading to congestive heart failure in ~5% of individuals. Methodology and pharmacogenetic studies for predicting which individuals are at high risk and subsequently the development of targeted and individualized cardioprotective plans are beginning to make progress. Here, we review current putative risk genes and variants, the strength of evidence for each genetic association and the interaction between risk genes, in the context of known clinical risk factors and potential novel cardioprotective strategies.

## 1. Introduction

Anthracyclines are a widely used class of chemotherapy drugs that induce cytotoxicity through double-strand DNA breaks. This class of chemotherapy is highly effective in multiple cancers including breast cancer, small cell lung cancer, acute lymphoblastic and myeloblastic leukemia, endometrial, gastric, liver, kidney, ovarian and thyroid cancers, Hodgkin and non-Hodgkin lymphoma, and Wilms’ tumor. The major limitation for use is cardiotoxicity manifesting as systolic dysfunction, in some cases leading to congestive heart failure. Anthracycline-induced cardiomyopathy and heart failure were first observed in clinical trials conducted in the 1960’s, but anthracyclines are still commonly used today in both adults and children. Given the oncologic success for so long, there is a clear need to be able to predict which patients will progress to congestive heart failure and how to protect these individuals.

Von Hoff et al. first demonstrated in 1979 that the probability of heart failure from anthracycline was dose dependent [1], which explained a significant proportion of the variability between patients. In this study, the overall incidence of heart failure was 2.2%, but the curve remained relatively flat below a cumulative dose up to 500 mg/m^2^. For patients receiving a cumulative dose > 500 mg/m^2^ the incidence of heart failure increased at a linear rate with > 50% incidence when the cumulative dose reached 800 mg/m^2^. A later study of 630 patients replicated the dose-dependent nature of anthracycline-induced heart failure, but the overall incidence was determined to be significantly higher at 5.1% [2]. In this study, the curve became linear at a cumulative dose of 450 mg/m^2^, at a cumulative dose of 550 mg/m^2^ the estimated incidence of heart failure was 26%, and older age (>65 years) appeared to be an important risk factor for heart failure. Nonetheless, due to the risk/benefit, it became a standard of care to administer anthracyclines at a “relatively safe” dose of up to 300 mg/m^2^. Ten years later, it also became apparent that moderate to low “safe” doses resulted in mild systolic dysfunction (left ventricular ejection fraction (LVEF) of < 50) in 26% of patients at six-month follow-up after the end of therapy [3].

The studies described above demonstrate that some patients can receive relatively high doses without heart failure and other patients experience heart failure at doses < 200 mg/m^2^. At this time, there is no approved clinical methodology to predict which of the significantly large proportion of patients with sub-clinical cardiac dysfunction will progress to symptomatic heart failure and cardioprotective strategies are limited. Therefore, in this review, we dissect the data on inter-individual variation with a focus on genetic studies, the interplay between the clinical risk factors and genetic variants and how the latest genetic findings will play a role in the development of future cardioprotective strategies.

## 2. Non-Genetic Risk Factors of Anthracycline-Induced Heart Failure

### 2.1. Age

Multiple studies of both males and females from clinical trial and real-world data have identified higher rates of anthracycline-induced cardiotoxicity (sub-clinical and congestive heart failure) in older individuals, including multivariate analyses which accounted for dose and other known risk factors [1,2,4,5,6,7,8,9,10]. Clearly, older age at time of treatment is a risk factor for anthracycline-induced cardiotoxicity, but where there is variability between studies is at what age. In studies of breast cancer, the consensus appears to be > 60–65 years old [2,4,10], although another study of breast cancer patients treated with epirubicin with 3–10 years follow-up showed that even patients > 40 years old at the time of treatment were at greater risk of congestive heart failure relative to younger patients, OR 2.19, *p* = 0.035 [6], and other studies only found significance at age > 50 years [9] and age > 70 years [5].

### 2.2. Gender

The combination of sex differences and age also form a significant part of inter-individual variation in anthracycline-related heart failure, which is demonstrated in both clinical and animal studies. For cardiovascular disease in general, prior to menopause, adult women experience less cardiovascular disease and maintain better cardiac function than men [11,12,13]. A similar trend is observed in the adult cancer population, although the data are less conclusive because most the data sets are from breast cancer patients with no male data for comparison. However, there are a small number of studies of anthracycline-induced heart failure in lymphoma patients, in which male sex is a significant risk factor (HR ranging 1.67–1.97, *p* = 0.019–<0.001) [7,14,15] and within breast cancer studies, older women (age > 60–65 years) are more at risk than younger women (HR ranging 2.25–3.20, *p* = 0.029–0.01) [2,4,10]. In contrast, pediatric studies of childhood cancer survivors suggest that young girls are at greater risk of anthracycline-induced heart failure compared to young boys [16,17,18,19,20,21,22], with the first report demonstrating an odds ratio of 3.2 for females versus males [22]. Data from rodent studies are in line with these findings, in that adult female mice and rats are much less susceptible to doxorubicin-induced decline in cardiac function and heart failure in comparison to males, but when ovariectomized, females become susceptible to doxorubicin-induced heart failure [23,24,25,26].

### 2.3. Race/Ethnicity

Patients in studies of anthracycline-induced cardiotoxicity have been predominantly white, but there are a handful of studies in which black cancer patients in the United States appear to be at higher risk of anthracycline-induced heart failure than the general population [27,28,29]. A retrospective study of 100 African-American patients with no previous history of cardiovascular disease, treated with doxorubicin at Howard University Hospital [28], showed the frequency of doxorubicin-induced heart failure (median follow-up time 1.3 years) to be almost three times higher than a reference study [29], (7% vs. 2.75%, *p* = 0.027), even after stratifying for cumulative dose. In this particular study, oncologists were already suspicious of a higher rate of cardiotoxicity in African-Americans, hence all were given a slower infusion of doxorubicin designed to reduce cardiotoxicity, leading the authors to suggest that the true frequency in the African American population may be even higher. However, a major limitation was lack of a matched population of other racial groups from the same institution, and the reference study chosen for comparison was published in 1973, and as described in the introduction, we know that the overall rate of doxorubicin-induced heart failure is actually higher than 2.75%. Nonetheless, the reported rate of heart failure in the Hasan study is still higher than that of the Swain study (overall incidence of 5.1%) [2] and a more recent study of 230 American and Afro-Caribbean patients treated with doxorubicin also reported a frequency of 7% [27].

Data on anthracycline-induced cardiotoxicity in the Hispanic population is extremely limited, but a small pediatric study of 46 patients with childhood acute myeloid leukemia, of which 23 (44%) were Hispanic, showed that decline in left ventricular systolic function was significantly worse among Hispanic patients compared with Caucasian patients [30].

### 2.4. Pre-Existing Conditions

Baseline LVEF [4,10], hypertension [31,32], coronary artery disease [32], and diabetes [6,32,33] have previously been identified as risk factors for anthracycline-related cardiotoxicity in some form, particularly when used concomitantly with trastuzumab [4,10]. It is not the goal of this review to discuss these factors in detail, many of which have an underlying genetic cause in their own right. It is our goal to stress the need for incorporation of actual causative genetic variants into risk prediction models of cardiotoxicity, rather than factors which may simply be proxies of the true underlying risk variants. Therefore, in the next sections, we detail the known genetic risk genes and variants, and their contribution to cardiotoxicity in the context of known clinical risk factors.

## 3. Genetic Variants Associated with Anthracycline-Induced Cardiotoxicity

### 3.1. Carbonyl Reductase 3 (CBR3)

The most well-replicated genetic association of doxorubicin-induced cardiotoxicity to date is the CBR3 V244M variant, rs1056892. The variation encodes a nucleic acid change of G>A resulting in the amino acid change of valine (GTG) to methionine (ATG), where A is the minor allele. The functional consequence of the allelic change was initially measured biochemically, where it was shown that the methionine variant had a 1.6-fold higher catalytic efficiency of NADP(H) than the valine variant, *p* = 0.013 [34] and the valine variant resulted in 1.7-fold higher catalytic efficiency of the anthracycline doxorubicin compared to the methionine variant, *p* < 0.05 [35,36] such that in the recombinant CBR3 protein, the valine allele synthesized 2.6 times more of the cardiotoxic doxorubicin metabolite (doxorubicinol) per unit of time [36].

Based on the functional data, one would hypothesize the valine (G allele) variant to be more frequent in patients with doxorubicin-induced cardiomyopathy/heart failure compared to patients who did not experience cardiotoxicity, and so far this seems to be the case. Multiple studies have now shown that the valine (G allele) is significantly more frequent in anthracycline-related cardiomyopathy in childhood cancer survivors [36,37] and declines in LVEF in early breast cancer patients [38,39]. In the Blanco study of childhood cancer survivors [37], at low-moderate anthracycline dose (<250 mg/m^2^), individuals with two copies of the valine (G allele) were at ~ threefold higher risk of cardiomyopathy compared to individuals with one or no copies of the G allele. However, at doses > 250 mg/m^2^, CBR3 genotype was not significant.

One might ask why this data is not incorporated into patient care plans, or at least into risk prediction models or prospective studies. There are some early signs, with a recent prospective study in which breast cancer patients due to receive doxorubicin were genotyped. In this study patients carrying at least one copy of the valine (G allele) experienced a significant reduction in LVEF at 6 months following initiation of doxorubicin compared to their pre-baseline study *p* < 0.001, with patients carrying two copies of the G allele exhibiting the largest reduction in LVEF (AG genotype versus GG genotype, 3.32% EF difference, 95% CI [−6.52, −0.12] *p* = 0.0397) [40].

#### 3.1.1. CBR3 Genetic Findings in Perspective

The risk variant in this case is the major (most common) allele, with a frequency in the GnomAD database of >140,000; individuals from different populations of ~63% and the frequency of “high risk” individuals with two copies of the G-allele would be ~40%. At a low-moderate dose of doxorubicin, we know that the frequency of heart failure and even sub-clinical change in LVEF is less than 40% (the Drafts study which assessed LVEF with MRI imaging, which is more accurate than echocardiography, showed 26% sub-clinical toxicity at 6 months post treatment [3]). One can only conclude that even if CBR3 V244M is truly a risk variant for anthracycline-induced cardiotoxicity, there are additional genetic risk genes and variants. This is also the case for several other risk variants described below and, to put into context, all genes discussed in this article and the frequency of the associated allele in the GnomAD database are shown in Table 1.

#### 3.1.2. Are There Other Variants in CBR3 That Could Account for Anthracycline-Induced Cardiotoxicity?

The initial study of CBR3 genetic variants and in vitro metabolism of anthracyclines tested a total of seven variants; allelic differences were identified for C4Y and V93I in addition to the V244M variant, and the largest reduction in metabolism of both doxorubicin and daunorubicin was observed for the double mutant (244M and 4Y) compared to wild-type enzyme and single variant preparations [35]. To date, publications of CBR3 genetic variants and cardiotoxicity have only covered the V244M variant. We can say that the C4Y rs8133052 variant is extremely rare (not present in the GnomAD database of > 140,000 individuals and frequency of 0.0004 in the PAGE study of 49,839 individuals of non-European ancestry [51]). Proving genetic association of such a rare variant would be challenging, but rare variants with a large effect size on cardiomyopathy are certainly possible and are observed as causative in genetic forms of dilated and hypertrophic cardiomyopathy [52]. Of further note, the metabolic studies of CBR3 variants also showed that both wild-type and variant recombinant CBR3 protein was able to metabolize doxorubicin 1.5-fold more efficiently than daunorubicin [35], suggesting that perhaps individuals deemed at higher risk of cardiomyopathy due to CBR3 variants might have less cardiotoxicity from daunorubicin.

#### 3.1.3. Could Differences in CBR3 Allele Frequency Contribute to Racial Differences in Anthracycline-Induced Cardiotoxicity?

The early papers on CBR3 genetic variants and anthracycline metabolism very quickly pointed out the variation in allele frequencies between different populations. The initial study of Bains et al. reports the 244M variant (referred to in the metabolic studies as the “mutant”) as being significantly more common in African Americans (52.5%) compared to Europeans, Chinese, and Japanese (30, 33, and 30% respectively). It is important to clarify at this point that the minor (or “mutant” methionine) allele in this case is not the risk allele for anthracycline-induced cardiomyopathy. It is the major (or “wild-type” valine) allele which is associated with anthracycline-induced cardiomyopathy. Hence, in this case the CBR3 V244M polymorphism does not at all explain the reported increased risk of anthracycline-induced cardiomyopathy in African American patients relative to other populations [28], as the allele associated with cardiomyopathy is less common in African Americans. Nonetheless, the CBR3 V244M variant may still be valid as a risk variant in African Americans, and as hinted at by the existence of the very rare C4Y variant in non-European populations in the PAGE study, there may be additional different rare missense variants within CBR3 that cause differential metabolism of anthracyclines, increase risk of cardiotoxicity and may be more frequent in some populations, and perhaps ought to be incorporated into clinical trials and prospective studies where treatment regimens that include anthracyclines.

### 3.2. Multiple Other Candidate Genes Are Involved in Intracellular Calcium Dysregulation, Anthracycline Metabolism, Transport and Generation of Reactive Oxygen Species Are Associated with Anthracycline-Induced Cardiotoxicity

More than 200 genes are biological candidates in the metabolism and transport of anthracyclines and, to date, five studies [38,41,42,45,53,54] have used the strategy of genotyping sets of 6 to 1931 single nucleotide variants mapping to 5 to 220 candidate genes. Of these studies, one replicated the CBR3 study above [38], two studies were unable to replicate [43,45], and three studies did not include the variant [41,42,54]. It is unclear if the failure of two other studies to replicate is due to the small to moderate sample sizes used (all were binary case/control analyses with the number of cases of cardiotoxicity ranging from 38 to 87 and the number of controls ranging from 118 to 363), or different definitions of cardiotoxicity and different types of cancer. Nonetheless, from the additional candidate gene studies, new associations have emerged, with more than one study finding association with the same variant (and same direction of association) as follows: RAC2 rs13058338 [42,43,44]; SLC28A3 rs7853758 [41,45], UGT1A6 rs17863783 [41,45]; and ABCB1 rs2235047 [38,45].

In the next section we describe the most recent findings from genome-wide association studies (GWAS) of anthracycline-related cardiotoxicity, which provide an unbiased screen of cardiotoxicity. Before we move to this section, we note that these unbiased approaches lend additional aid in replication of the candidate genes above. For example, the GWAS study of Serie et al. [39], which used a quantitative analysis of the decline in LVEF in patients with breast cancer, also observed association *p* < 0.05 with the CBR3 V244M variant, and with ABCB1 rs2235047, and showed a trend for association with UGT1A6 rs17863783 *p* = 0.078.

### 3.3. Genome-Wide Association Studies Have Identified New Loci That Are Associated with Anthracycline-Induced Cardiotoxicity and Replicated Some Candidate Gene Studies

Five published GWAS studies [39,46,47,48,49] have identified several putative risk genes and variants for anthracycline-induced cardiotoxicity. The advantages of these designs are their unbiased nature and capability to identify risk loci, irrespective of gene function. The downside of such an approach is the need for multiple testing corrections due to the large number of variants tested. Of course, this could be somewhat offset by a large sample size, and herein lies the problem, particularly with pharmacogenomic studies in which sample size is notoriously smaller than that of studies which simply associate a disease state with genetic variants [55]. A perfect illustration would be that of CBR3 V244M, a “replicated” variant for anthracycline-induced heart failure. Of the five published GWAS studies, CBR3 was not reported amongst the top hits for genome-wide significance or even “suggestive” evidence for genome-wide significance. Two of the GWAS studies did, however, report association at *p* < 0.05 in the same direction [39], and association of this variant was not discussed/disclosed in the other GWAS. Notably, the study of Serie et al. [39] was relatively large (*N* = 1191), using DNA samples from a phase III clinical trial, but the study of Wang et al. [49] was not truly an independent replication because there was 84% patient overlap with the candidate gene study of Blanco et al. [37]. These data could be interpreted in one of two ways. Firstly, the CBR3 association did replicate and is likely a true risk variant that could be of use in the clinic. Secondly, there may be other risk genes that would not have been investigated with a candidate gene approach that are more significant and/or have a larger effect size on the outcome of anthracycline-related cardiotoxicity. Hence, in the sections below we examine the evidence for the top hits from each GWAS.

#### 3.3.1. Retinoic Acid Receptor Gamma (RARG)

One of the key traits for GWAS studies is that the most significant variants are nearly always non-coding variants, such that the additional fine mapping required to identify the most likely “causative/functional variant” or even the correct locus can be very challenging. Interestingly, in the first published GWAS of anthracycline-induced heart failure, the most significant variant was a missense variant in RARG (Ser427Leu TCG>TTG, rs2229774) [46] and there was a trend for association of the same variant with CHF, *p* = 0.07 in the GWAS study of Serie et al. [39]. In the initial Aminkeng GWAS [46], fine mapping by imputation identified three intronic variants within RARG with *p*-values of equal or greater significance and effect size (*p* ranging 1.7 × 10^−6^ to 4.1 × 10^−6^ and OR 7.0–7.5) to rs2229774 (*p* = 5.0 × 10^−6^ and OR = 7.0). The intronic variants were in moderate (r^2^ = 0.55) to high (r^2^ = 0.84) linkage disequilibrium with rs2229774, such that it is not clear as to which variant is truly causative/functional, but given that the initial variant changed an amino acid, the authors made the natural and obvious choice to continue functional validation efforts with the Ser427Leu variant. In these experiments, using a myoblast cell line from rat, the wild-type allele (427Ser) significantly reduced the expression of Top2b and the 427Leu allele that was associated with increased risk of heart failure showed only weak reduction of Top2b. This is important functional evidence because it is already known that doxorubicin binds to Top2b and that in mice, cardiomyocyte-specific deletion of Top2b is protective against doxorubicin-induced heart failure [56].

Identification of a human variant that prevents reduction of TOP2b expression is valuable evidence for transfer of knowledge from the bench to the bedside, and in addition, the Aminkeng study did include a replication group and meta-analysis showing association of rs2229774 427 Leu variant with increased risk of heart failure. However, an independent and much larger GWAS study of 3431 patients with breast cancer found significant association (*p* = 4.1 × 10^−6^) but with the opposite allele. This is highly relevant for transfer to the clinic, and we note here that there are many examples in the literature with a flip-flop effect in which the opposite alleles of the same variant are associated with increased risk of disease, which occur when the investigated variant is correlated with the true causative/functional variant through interactive effects or linkage disequilibrium which differs between populations [57,58,59,60]. Therefore, what may be required to truly move this gene towards clinical guidance is additional fine mapping and exon sequencing across different populations of patients with anthracycline-induced heart failure. Although rs2229774 is the most common missense variant in the GnomAD database, there are >200 missense, frameshift, and loss-of-function variants at lower allele frequencies (<1%). A GWAS analysis would be underpowered to detect associations with such rare variants, but it remains possible that (1) other rare missense alleles from multiple cases within a GWAS happen to be present on the same chromosome as the 427 Leu variant, and (2) other rare RARG amino acid changes also result in reduced binding to RARG and hence greater cardiotoxicity.

#### 3.3.2. Intergenic Variant rs28714259

At the time of writing, the most stringent GWAS of anthracycline-induced heart failure used DNA from breast cancer patients in three phase III clinical trials [47]. The discovery sample of patients in the E5103 trial used a binary analysis of congestive heart failure in 68 cases vs 987 controls, yielding nine variants associated at *p* < 10^−5^ with increased risk of congestive heart failure (HR = 2.1–2.7) from which they selected two variants (an intronic variant rs6883259 in ADCY2 and an intergenic variant on chromosome 15, rs28714259) for replication in a second sample of 47 cases and 883 controls from the E1199 trial. Of these two loci, rs28714259 met the criteria for replication, *p* = 0.04, OR = 1.9 (95% CI, 1.0–3.7) with association of the same allele. Association of the same allele was confirmed within a third sample of 24 cases and 298 controls. Nonetheless, using some very stringent criteria, the Schneider study identified rs28714259 to be associated with anthracycline-induced heart failure in three independent sets of patients. A downside of this stringent approach to replication was that true variants could potentially have been discarded, and that the top associated variant mapped to an intergenic region such that functional validation studies will be more challenging.

#### 3.3.3. PR Domain Containing 2 with ZNF Domain (PRDM2)

Due to the relatively small proportion of patients with symptomatic heart failure from anthracycline and need for adjustment for multiple testing in GWAS, two of the published GWAS used an alternative approach to improve statistical power by using a quantitative approach that tested for association of genetic variants with the maximum decline in LVEF [39,48]. Of course, there are caveats to the quantitative approach, mainly the variability and lack of sensitivity in echocardiography and serial echocardiographic measurements. Using this approach, with 385 patients from the Vanderbilt Biobank as a discovery set and an independent replication set of patients, Wells et al. [48] identified the PRDM2 variant rs7542939 as a risk variant for anthracycline induced decline in LVEF (*p* = 6.5 × 10^−7^ in meta-analysis of both data sets). At the time of writing, there are no further replications or functional validation studies of this locus, although there is biological plausibility. PRDM2 is known to be critical for repair of double-strand DNA breaks [61] and impairment of this mechanism was shown to exacerbate doxorubicin-induced cardiotoxicity in mice [62].

#### 3.3.4. CUGBP Elav-Like Family Member 4, CELF4 rs1786814

CELF4 was first associated with anthracycline-induced cardiomyopathy in a GWAS study of childhood cancer survivors [49] by the same group that first identified CBR3. In fact, the GWAS study had 84% overlap with the CBR3 study set. The association of CBR3 replicated (*p* = 0.03) as one might expect with such a large overlap, but did not reach genome-wide association, likely due to the moderate sample size (cases *N* = 112, controls *N* = 219). Indeed, in this study, no variants reached genome-wide significance or were marginally close to this level of significance. However, the authors intelligently used the dataset to perform a two-step analysis of gene-environment interaction. In the first step, they selected variants for which suggestive main effects were observed (~1000 variants, *p* < 0.004). In the second step, they tested the limited variant set for gene-environment interaction, by the extent of modification of the association between anthracycline dose and risk of cardiomyopathy. In this analysis, individuals with the CELF4 rs1786814 GG genotype, who received high dose of anthracyclines (>300 mg/m^2^) were at 10-fold higher risk of cardiomyopathy (*p* < 0.001) compared to those with the GA/AA genotype and low to moderate dose (<300 mg/m^2^). For individuals with the GA/AA genotypes (~34%), the risk of cardiomyopathy did not increase with increased anthracycline exposure. The study replicated this finding in a small replication set of 54 patients with cardiomyopathy, *p* = 0.046.

CELF4 is a known splicing regulator of cardiac troponin T (TNNT2), which plays an essential role in calcium signaling in cardiac muscle, and rare genetic variants in TNNT2 are associated with familial dilated and hypertrophic cardiomyopathy [52]. Given these data, the authors performed further functional analyses, in which the GG risk genotype was found to be more frequent in adult individuals whom expressed both the adult and embryonic TNNT2 splice variants in their heart tissue, the coexistence of which is associated with decreased ventricular pumping [63,64].

To date no other studies have published replication or association with the CELF4 rs1786814 variant, but searching through the GWAS data of Serie et al. [39] (described in more detail in the section below) did identify a different variant to be associated with doxorubicin-related decline in LVEF, *p* = 0.0008 (unpublished data), which maps in the same intron and within 30 kb of rs1786814. These two variants are not in linkage disequilibrium (not inherited together) and therefore, if both associations are correct, there may be multiple independent risk variants in the CELF4 intron one that are possibly associated with aberrant splicing of cardiac troponin, and fine-mapping studies of this gene are justified.

#### 3.3.5. Transient Receptor Potential Cation Channel Sub-Unit Six (TRPC6)

TRPC6 was identified in a GWAS study of 1191 patients in the N9831 clinical trial, using a quantitative analysis of maximum decline of LVEF [39]. The N9831 trial was designed to test the efficacy of the addition of trastuzumab to doxorubicin and paclitaxel in patients with early breast cancer. Approximately one third of patients received doxorubicin, cyclophosphamide, and paclitaxel, while two thirds of patients received doxorubicin, cyclophosphamide, paclitaxel, and 12 months of trastuzumab. Therefore, it is possible that genetic associations with decline in LVEF were actually associations with trastuzumab-related cardiotoxicity. For discovery purposes, the GWAS reported initially on the patients who received all treatments (doxorubicin, cyclophosphamide, paclitaxel, and trastuzumab (*N* = 800), identifying six independent loci with significance of *p* < 1 × 10^−5^ (TRPC6, RAB22A, LDB2, BRINP1, LINC01060, and an intergenic variant on chromosome 6). Post-hoc analysis was performed separately for each of these loci in the 391 patients who did not receive trastuzumab and for all 1191 patients combined under the assumption that associations resulting from chemotherapy might increase in significance. Only TRPC6 rs77679196 showed any trend for association in the smaller set of patients who did not receive trastuzumab (chemotherapy only), *p* = 0.06, and significance increased when all patients were combined (*p* = 1.63 × 10^−6^, beta = −6.87) suggesting that cardiotoxicity associated with this variant was the result of doxorubicin and not trastuzumab. Interaction analyses suggested that one of the top loci (RAB22A, rs707557) was the result of the addition of trastuzumab to doxorubicin and it was undetermined if cardiotoxicity associated with the remaining four loci was the result of chemotherapy, trastuzumab or a combination of both (unpublished data).

A subsequent study by the same group [50], replicated the association of TRPC6, this time using a case-control analysis with anthracycline-induced congestive heart failure as the outcome (Cases *N* = 38, Controls *N* = 984). In this study, the authors performed several levels of analyses which provided evidence for multiple risk variants at TRPC6, firstly suggesting that common non-coding variants within intron 1 and the 5’flanking region of TRPC6 increase risk of cardiotoxicity by their association with increased TRPC6 expression in heart tissue, and secondly that both common and rare missense variants at TRPC6 may increase risk of cardiotoxicity by gain-of-function. The significance of these findings is that inhibition of TRPC6 may be a novel cardioprotective therapy, especially for individuals with TRPC6 risk variants. The therapeutic potential was validated in vitro, by demonstration that iPSC-derived cardiomyocytes were protected from doxorubicin-induced apoptosis by pre-treatment with the TRPC6 inhibitor, GsMT×4, and in vivo, by demonstration that GsMT×4 also protected mice from doxorubicin-induced decline in LVEF, global longitudinal strain and fibrosis [50].

A final point from the Norton study [50] especially highlights the need for mapping risk variants in different populations, but also to not rely completely on imputation for fine mapping of GWAS data. Sequencing of the 38 cases with doxorubicin-induced heart failure identified a very rare missense variant (N338S) in one of only four African-American individuals. The patient carrying the 338S variant should have been at low risk for cardiotoxicity (age 32 years old at time of chemotherapy and baseline LVEF of 62, and dose in this dataset was kept to under 350 mg/m^2^). The N338S variant is extremely rare and appears to be unique to individuals of African ancestry, with a frequency of 0.02% in Africans and 0% in European ancestry (frequencies from GnomAD database of > 140,000 individuals). To put into context, this variant occurred in one of four (25%) African-Americans with doxorubicin-induced heart failure, and could potentially account for a significant proportion of risk in individuals of African ancestry. Preliminary data published as an abstract at the American Society of Human Genetics 2021 demonstrated in vitro that the rare TRPC6 338S variant results in increased TRPC6-specific electrical current and increased calcium influx compared to the wild-type N338 variant when under activation [65]. The same study also demonstrated that treatment of both wild-type and mutant cells with doxorubicin resulted in increased calcium influx (with highest increase in the mutant cells) that was prevented by pre-treatment with the TRPC6-specific inhibitor BI749327, suggesting that BI749327 is a potential cardioprotective therapy for both patients carrying TRPC6 risk variants and those without. Aside from cardiotoxicity, TRPC6 is already well known to be an important contributor to pathological cardiac remodeling [66,67] and when administered orally in a mouse model of hemodynamic stress, BI749327 was able to improve cardiac function and reduce chamber dilation and fibrosis [68].

### 3.4. Are Cardiotoxicity Risk Genes Linked via the Same Mechanisms?

**Figure 1:**
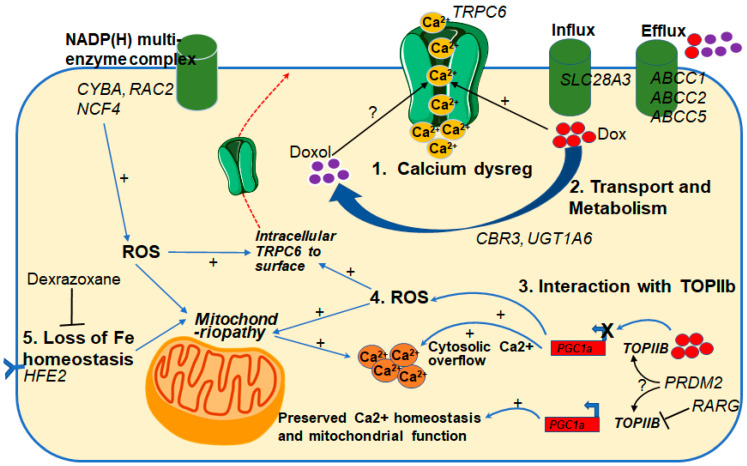
Multiple putative risk genes and pathways of anthracycline-related cardiotoxicity. Putative risk genes are shown in uppercase italics within their proposed pathway of cardiotoxicity: (1) Calcium dysregulation; (2) Transport and metabolism; (3) Interaction with TOP2B; (4) Generation of Reactive Oxygen Species (ROS); (5) Iron homeostasis. Arrows mark pathways and potential interaction between pathways of cardiotoxicity. For example, generation of ROS and loss of iron homeostasis results in mitochondriopathy and cytosolic calcium overflow, but interaction of anthracycline with TOP2B also results in down-regulation of PGC1a and subsequent cytosolic calcium overflow. Increased TRPC6 expression or activity is influenced by genetic variants and results in increased calcium influx, but doxorubicin alone may also increase TRPC6 activity. Genetic variants in anthracycline transport and metabolism-related genes will affect the level of intracellular anthracyclines and their different metabolites and potentially increase (+) or decrease TRPC6 activation. ?, effect of metabolite or variant not known.

Putting the putative risk genes into context and determination of how they may be linked may be an important strategy for determining which genes to target for cardioprotection (Figure 1). For example, risk variants in TRPC6 likely result in either gain-of-function or increase expression and therefore increased intracellular calcium levels. Therefore, inhibitors of TRPC6 may serve as cardioprotective therapies for individuals with TRPC6 risk variants, but what about individuals with risk variants in other genes?

In vitro data from our group has shown that a TRPC6 gain-of-function variant (N338S) results in increased calcium influx through TRPC6 when under activation (not at baseline), but also that even for wild-type TRPC6, calcium influx is increased in the presence of doxorubicin. As shown in Figure 1, doxorubicin is metabolized by CBR3 into doxorubicinol, and doxorubicinol is more cardiotoxic than doxorubicin [69]. The reason for increased cardiotoxicity of doxorubicinol is not known, and neither is the effect of doxorubicinol on TRPC6. One hypothesis is that doxorubicinol is more cardiotoxic than doxorubicin because it increases TRPC6-mediated calcium influx more than doxorubicin. If this hypothesis is true, then individuals with the CBR3 V244 risk variant that is shown to metabolize doxorubicin into doxorubicinol ~2.6 times faster than the M244 variant may also be candidates for cardioprotection by TRPC6 inhibition. If the hypothesis is shown not to be true and CBR3 and TRPC6 mechanisms of cardiotoxicity are completely unrelated, then one would suggest TRPC6 inhibition for one set of individuals and CBR3 inhibition for those with CBR3-specific risk variants. Alternatively, there is also evidence that doxorubicinol participates in the proposed mechanism of iron-related cardiotoxicity [69], in which case, the FDA-approved drug and iron chelator dexrazoxane may also be a preferred cardioprotective therapy for individuals carrying CBR3 risk variants.

Generation of reactive oxygen species is a major proposed mechanism of doxorubicin-induced cardiotoxicity [70], and genetic risk variants are mapped to several genes involved in the NADP(H) multi-enzyme complex including *CYBA*, *RAC2*, *NCF4*, which leads to the generation of ROS (Figure 1). Similarly, risk variants also map to RARG, an inhibitor of TOPIIb. The RARG genetic variants associated with cardiotoxicity lead to reduced inhibition of TOPIIb. When TOPIIB is not inhibited, it binds to doxorubicin and together, this complex binds to the promoter of PPARGC1A to prevent transcription. PPARGC1A has antioxidant properties and when its transcription is reduced, there is an increase ROS, mitochondrial death [56], and cytosolic calcium overflow [71]. Research in the field of chronic kidney disease (itself a risk factor for heart failure) has shown that ROS lead to increased surface expression of TRPC6, which must be at the surface to be active [72]. If this is also the case in cardiomyocytes, it may be possible that inhibition of TRPC6 is a potential cardioprotective therapy for individuals carrying risk variants in the RARG and NADP(H) complex genes.

### 3.5. From Genetic Risk Variants to Cardioprotection, Chemosensitization and Anti-Tumor Properties

As we discover more of the genes and pathways relating to doxorubicin-induced cardiotoxicity, and begin to assess each gene as a therapeutic target for cardioprotection, we must also consider the effect in relation to the tumor. For example, the only FDA-approved therapy for cardioprotection against doxorubicin currently is the iron chelator dexrazoxane, but its use has been limited and there are concerns of interference with anti-tumor efficacy [73]. In the current literature, at least two of the cardiotoxicity risk genes discussed may have both cardioprotective and anti-tumor properties.

Firstly, the CBR-mediated doxorubicin-metabolites, including doxorubicinol, are known to be effluxed at a faster rate through the ABC-transporter genes [69] which are also cardiotoxicity risk genes (Figure 1). This means that individuals with higher rates of doxorubicin metabolism into doxorubicinol (such as those with the CBR3 244V variant), may also have reduced efficacy in relation to the tumor. This hypothesis is based on in vitro evidence in which cancer cell lines that rapidly metabolized doxorubicin and had higher expression levels of CBR3 were also more resistant to doxorubicin-cytotoxicity than slow-metabolizing cancer cells [74]. Therefore, therapies that inhibit the CBR genes may be both cardioprotective and increase chemosensitivity.

Secondly, dysregulation of calcium homeostasis has been observed in multiple pathological conditions including cardiac remodelling and heart failure [75,76,77,78] and tumorigenesis [79]. Specifically, transient receptor potential channels (including TRPC6) which are key regulators of intracellular calcium homeostasis are also involved in the main processes of metastasis such as migration, invasion, and tumor vascularization [80]. There are now multiple in vitro studies that have reported that inhibition or genetic knock-down of TRPC6 significantly reduces cancer cell migration and proliferation [81], suggesting that TRPC6 inhibition may be beneficial both in terms of cardioprotection from doxorubicin and anti-tumor efficacy.

## 4. Summary

Several genes (*CBR3*, *UGT1A6*, *RARG*, *SLC28A3*, *ABCC1*, *ABCC2*, *ABCC5*, *CYBA*, *RAC2*, *NCF4*, *HFE2*, *CELF4*, *PRDM2*, *TRPC6*, and intergenic variants) have now emerged as risk loci for anthracycline-related cardiotoxicity, but more detailed/fine mapping, replication, and functional validation is required to determine the exact causative/functional variant(s) for each gene and to move these variants from the laboratory to a clinical risk model that can be incorporated into patient records and used prospectively. Several of the known risk variants have some degree of functional validation that contributes to the putative mechanisms of cardiotoxicity, including dysregulation of intracellular calcium, transport and metabolism, interaction with TOP2B, generation of ROS, and iron homeostasis, with multiple genes in previously thought different mechanisms converging towards dysregulation of calcium homeostasis. Non-coding risk variants have also been identified by GWAS and replicated in multiple cohorts. These variants should also be incorporated into future risk models, but determination of their function by which gene(s) that they regulate, thereby allowing personalized therapy, will be more challenging, whereas risk loci mapping to coding genes with functional data are pointing towards CBR, TOP2B, and TRPC channel inhibitors as potential cardioprotective therapies.

## Figures and Tables

**Table 1:** Genes and variants associated with anthracycline-induced heart failure and the frequency of each associated variant in sequence data from > 140,000 individuals in the GnomAD database. Associated allele is shown in bold.

Gene ID	Function	Variant ID	Associated Allele [Reference]	Frequency of Associated Allele in GnomAD Database
*CBR3*	Carbonyl reductase 3 catalyzes the reduction of a large number of carbonyl compounds to their corresponding alcohols including doxorubicin to doxorubicinol and daunorubicin to daunorubicinol.	V244M rs1056892 G>A	V244 **G**TG [34,36,37]	0.6309
*UGT1A6*	An enzyme of the glucuronidation pathway that transforms small lipophilic molecules, such as steroids, bilirubin, hormones, and drugs, into water-soluble, excretable metabolites.	V209V rs17863783 G>T	V209 GT**T** [41]	0.0394
*RAC2*	Member of Ras superfamily of small guanosine triphosphate (GTP)-metabolizing proteins. Involved in the generation of reactive oxygen species.	rs13058338 T>A	**A** [42,43,44]	0.1960
*SLC28A3*	Solute carrier family 28 member 3 regulates multiple cellular processes, including vascular tone and metabolism of nucleoside drugs.	L461L rs7853758 C>T	L461 **C**TG [41,45]	0.8429
*ABCB1*	ATP-dependent drug efflux pump for xenobiotic compounds. Responsible for decreased drug accumulation in multidrug-resistant cells and often mediates the development of resistance to anticancer drugs.	rs2235047 A>C	**C** [41]	0.0894
*RARG*	Retinoic acid receptors bind to DNA regulatory sequences termed retinoic acid response elements (RAREs) and transcriptionally co-regulate downstream gene expression in response to their agonist, all-trans retinoic acid (ATRA). RARG can both activate and repress transcription in response to ATRA.	Ser427Leu rs2229774 C>T	L427 T**T**G [46]	0.0728
Intergenic *15q11.2*	Unknown	rs28714259 G>A	**A** [47]	0.1810
*PRDM2* (SNP located ~40 kb downstream)	*PRDM2* is critical for BRCA1-dependent repair of DNA double-strand breaks and is also a transcriptional regulator of heme-oxygenase-1, which plays a role in protection from oxidative stress.	rs7542939 A>G	**G** [48]	0.1187
*CELF4*	RNA-binding protein. Activates exon 5 inclusion of cardiac isoforms of *TNNT2* during heart remodeling. Promotes exclusion of both the smooth muscle (SM) and non-muscle (NM) exons in actinin pre-mRNAs.	rs1786814 G>A	**G** [49]	0.8443
*TRPC6*	Transient receptor potential cation channel subfamily C member 6 is a receptor-activated calcium channel in the cell membrane involved in cardiac remodeling.	rs77679196 G>A N338S rs767086724 A>G	**A** Ser338 A**G**C [39,50]	0.0176 0.00002
